# Incorporation of Nitinol (NiTi) Shape Memory Alloy (SMA) in Concrete: A Review

**DOI:** 10.3390/ma18194458

**Published:** 2025-09-24

**Authors:** Muhammed Turkmen, Anas Issa, Omar Awayssa, Hilal El-Hassan

**Affiliations:** 1 Department of Civil and Environmental Engineering, United Arab Emirates University, Al Ain 15551, United Arab Emirates; 700041940@uaeu.ac.ae; 2 College of Engineering and Technology, American University of the Middle East, Kuwait City 54200, Kuwait; anas.issa@aum.edu.kw; 3 Department of Chemical and Petroleum Engineering, United Arab Emirates University, Al Ain 15551, United Arab Emirates

**Keywords:** nitinol, shape memory alloy, concrete reinforcement, superelasticity, structural resilience, construction materials

## Abstract

Incorporating Nitinol (NiTi) shape memory alloy (SMA) into concrete structures has gained significant attention in recent years due to its ability to enhance the properties of concrete. This review paper illustrates the history of NiTi SMA and its use in various civil engineering structural applications. A detailed analysis of the existing literature and case studies offers perspectives on the possible applications, benefits, and prospects of utilizing NiTi SMA to reinforce and strengthen elements in concrete structures. The study examined publications on the internal usage of NiTi SMA in concrete and cement-based matrices as an embedded element, including fibers, bars, cables, wires, powder, and strands. In addition, superelastic and shape memory forms of NiTi were considered. It was concluded that the superelasticity of NiTi aided in energy dissipation from impact or seismic events. It also improved the re-centering performance and deformation capacity and reduced residual stresses, strains, and cracks. Conversely, the SMA effect of NiTi helped bridge cracks, recover the original shape, and induced prestressing forces under thermal activation.

## 1. Introduction

As the construction industry progresses and develops rapidly, it is expected to accommodate more advanced, resilient, and ductile designs against earthquakes, blasts, and natural phenomena. A potential emerging solution is smart materials, which are currently adopted by various industries to enhance specific products or systems’ behavior. Of these materials, shape memory alloys (SMA) are innovative materials that possess outstanding properties and can enhance the behavior of structural elements. SMAs display distinctive traits that render them favorable for various engineering applications, especially their superior energy dissipation capacity compared to conventional metallic materials. The Swedish physicist Arne Ölander identified the first SMA in 1932 within gold-cadmium alloys. In 1938, Greninger and Mooradian observed a similar feature in copper-zinc (CuZn) and copper-tin (CuSn) alloys. However, the name “shape memory alloy” was not created until Vernon used it, referring to his polymeric dental material [1]. Typically, SMAs are iron, copper, niobium, or nickel-based [2,3,4]. The application of SMA is not only limited to civil engineering but also extends to various fields, such as automobile and mechanical engineering applications, automotive, aerospace, mini actuators and micro-electromechanical systems, robotics, biomedical, and clothing/fashion industries [1].

Due to their high cost, SMAs were mainly used in the past in small quantities as connectors, isolators, braces, and retrofitting elements to improve the performance of steel structures under seismic load [5]. Their utilization expanded to reinforce and strengthen elements in concrete members, with findings revealing exceptional improvement in performance, including better resistance to crack propagation, less deflection, and improved ductility [3,6,7]. It has also enhanced the performance of structures under earthquake loads [8]. However, their use is still limited to small buildings and elements [5]. Indeed, a reduction in the cost of SMA would promote its wider use in structural and civil engineering applications.

Among the SMA mentioned earlier, a nickel-based SMA, called Nitinol (NiTi), is the most frequently used in civil engineering applications owing to its remarkable properties compared to other SMA [9]. Research related to NiTi applications in concrete has lately become a research trend. Therefore, this paper serves as a literature review of the use of NiTi in structural concrete applications and familiarizes researchers with the work achieved while pinpointing potential gaps and suggesting new avenues for future investigation. In this context, this study summarizes 71 research papers that investigate and explore the structural applications of NiTi SMA embedded in concrete and cement-based composites, i.e., as a reinforcing and strengthening material. It is worth noting that only research papers covering the structural applications of NiTi in concrete were utilized in this literature review. Other applications of NiTi, like damping devices, bracings, bolts, anchors, and uses in steel structures, have been excluded and are not within the scope of this review.

## 2. Background on SMAs

### 2.1. Overview of SMAs

**Figure 1:**
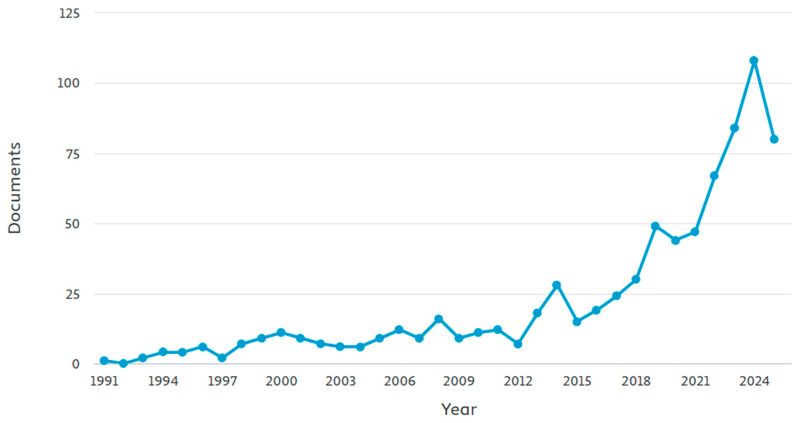
Number of SMA-oriented papers versus year of publication (civil engineering applications).

Figure 1 tracks the growth of the number of papers featuring SMA published annually from 1991 to 2025. Papers steadily, but slowly, increased from 1991 to 2006. In 1991, Graesser and Cozarelli [10] were the first to use SMA in civil engineering applications as seismic isolators due to their great damping ability. Beyond 2006, a considerable acceleration in the publications of SMA papers was noted, with consistent peaks and troughs indicating periodic fluctuations. In the past five years, SMA has become a widely researched topic in civil engineering. It is imperative to highlight that the drop in 2025 is unrealistic and should not be considered because of the incomplete data input for that year when the graph was generated. The upward trend in SMA research demonstrates its growing importance across civil engineering applications. This highlights the material’s versatility and potential for innovative applications in civil engineering and beyond.

**Figure 2:**
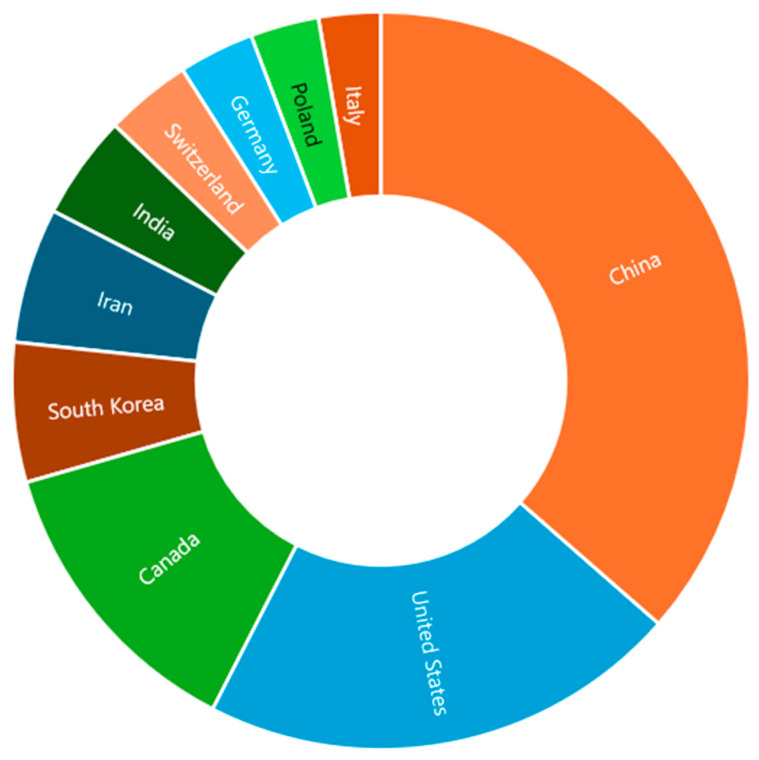
Percentage Distribution of SMA publications related to civil engineering around the world.

Furthermore, Figure 2 presents the distribution of SMA papers across different countries. China stands out as the leading country in the field of SMA research in civil engineering, followed by the USA and Canada in second and third place, respectively. Other notable countries include South Korea, Iran, India, Switzerland, Germany, Poland, and Italy. As global interest continues to increase, collaboration in research efforts could further accelerate and enhance advancements in SMA technologies. Moreover, exploring how SMA innovations can address emerging challenges in sustainability and resilience will open new opportunities for development in civil engineering and other industries.

### 2.2. Characteristics of SMAs

Shape memory alloys are a class of metallic materials characterized by their ability to return to a predetermined shape upon removing an imposed mechanical load or applying heat. This unique behavior is governed by a reversible phase transformation, which results in distinct changes in the material’s microstructural crystal arrangement. SMA predominantly exists in a less rigid martensite phase and a more robust austenite phase. Temperature variations primarily dictate the phase transition. At elevated temperatures, it assumes the austenite phase, exhibiting superelastic properties, allowing it to undergo significant deformation under applied stress and recover its original configuration upon unloading. Conversely, SMA transitions to the martensite phase at lower temperatures, manifesting the shape memory effect, where deformation is retained until the material is reheated. At this point, it reverts to its original shape.

**Figure 3:**
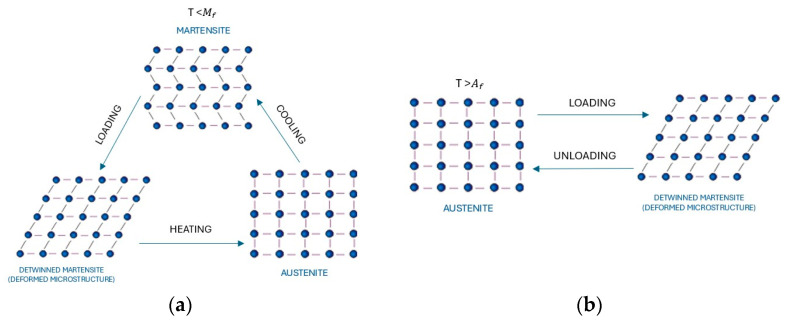
Microstructural behavior of SMA: (**a**) Superelasticity, (**b**) shape memory effect.

**Figure 4:**
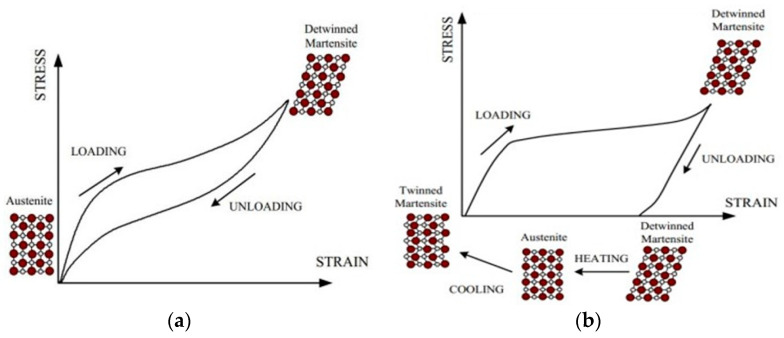
Stress–strain curve of SMA: (**a**) Superelastic behavior, (**b**) shape memory behavior [12] (red atoms represent Nickel; white atoms represent titanium).

Figure 3 and Figure 4 visualize the microstructural behavior and stress–strain relation of shape memory alloys in superelastic and shape memory effect states, respectively. It is essential to highlight that the range of temperatures of the phase transformation from austenite to martensite or the opposite changes depending on the chemical composition of the SMA. In addition, the transformation temperatures can be modified using processing techniques, such as cold drawing, as indicated in Kim et al. [11]. In this context, SMAs can undergo considerably recoverable large strains up to 8% due to their self-centering, energy dissipation, and shape recovery abilities. Additionally, SMAs have good corrosion resistance, unlike steel. These intrinsic properties position them as highly advantageous for strategic utilization in regions characterized by earthquake activity. Recent experimental and numerical research has represented several SMA uses in civil engineering for performance enhancement of structures under earthquake loads [8].

## 3. Nitinol: A Promising SMA

### 3.1. Overview of Nitinol

**Figure 5:**
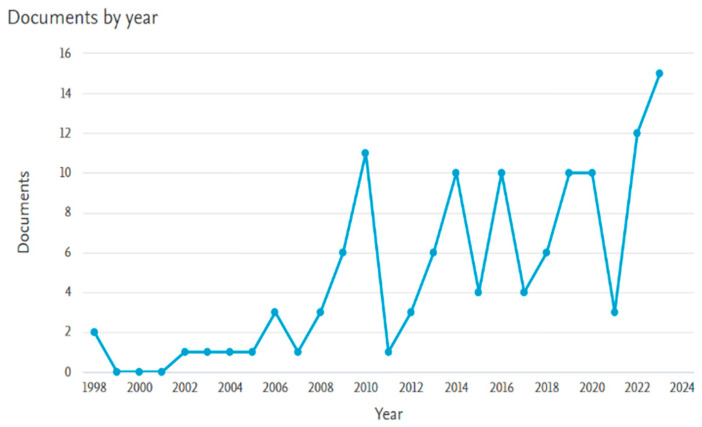
Number of NiTi-oriented papers versus year of publication (reinforcing and strengthening applications).

**Figure 6:**
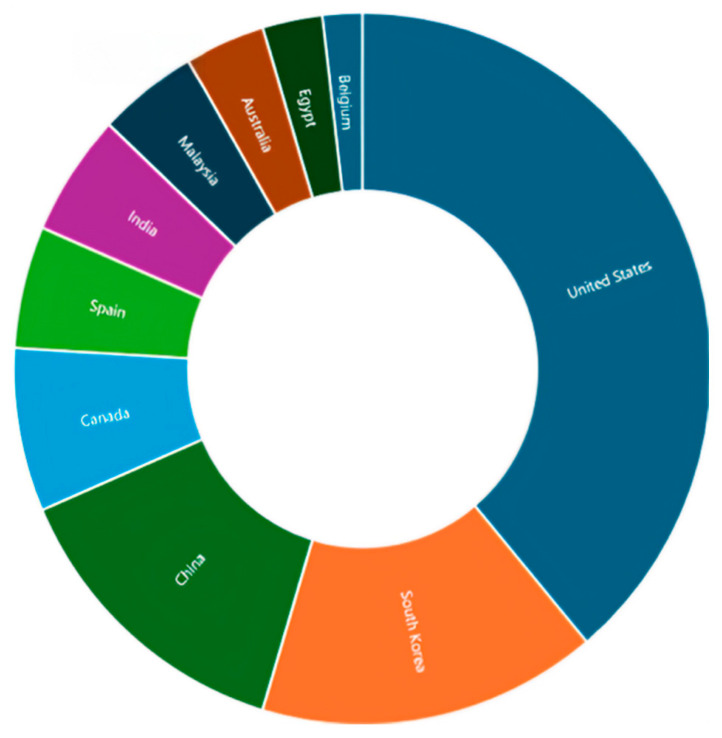
Percentage distribution of NiTi publications related to civil engineering around the world (limited to the top 10 countries).

NiTi, short for Nitinol, is a distinctive type of SMA. As implied by its name, it is an alloy made of nickel and titanium, with almost equal atomic percentages of both metals. The name Nitinol stands for Nickel (Ni) Titanium (Ti) Naval (N) Ordnance (O) Laboratory (L). It is derived from the name of the laboratory where Buehler and Wiley first discovered it during their experiments in 1965 [13]. Nitinol’s unique properties paved the way for its usage in various fields, such as aerospace, heating and ventilation, safety and security, automation and control, chemical processing, electronics industries, automotive, appliance/and robotics, where Buehler and Wiley first discovered it during their experiments in 1965 [13]. Additionally, it was used in civil engineering applications owing to its highly advantageous thermomechanical and thermo-electrical properties, exceptional superelastic behavior, strong corrosion resistance, reduced vulnerability to temperature changes, and remarkable fatigue endurance [9,13]. Figure 5 illustrates the published works on the use of NiTi in civil engineering applications (i.e., reinforcing and strengthening) over the years, while Figure 6 presents the distribution of these publications across different countries. Although the use of NiTi in civil engineering started off at a low rate early on, its use since 2010 has increased despite some fluctuations over the years. In this regard, the United States, South Korea, and China have authored most of the publications in this research field.

**Figure 7:**
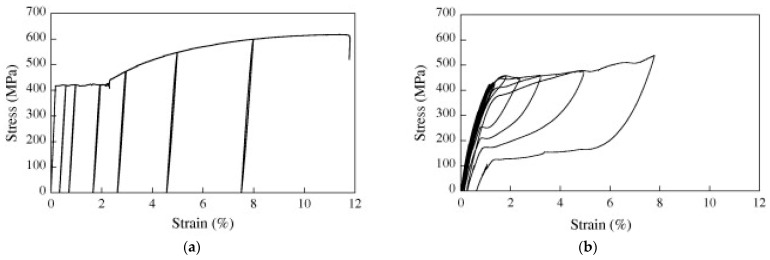
Cyclic stress–strain curve of (**a**) steel and (**b**) NiTi SMA [14].

Molod et al. [5] compared the properties of NiTi to those of steel. The authors displayed comparable yield and ultimate strength, higher recoverable elongation, and better corrosion resistance in favor of NiTi. In other works [14], the authors investigated the performance of SMA-reinforced concrete elements subjected to monotonic and cyclic loading and compared them to counterparts reinforced with steel. The stress–strain curves of Figure 7 demonstrate that NiTi was strained to 7.7% with 500 MPa ultimate stress. On the other hand, steel ruptures at 11.5% with 600 MPa ultimate stress. The cyclic test indicated a residual strain of less than 0.5% for the NiTi and almost 7.5% for the steel at the same loading. Moreover, a significant reduction in steel’s strain capacity was observed compared to the negligible loss in NiTi. The testing indicated that NiTi displayed superior performance in the tensile cyclic test and was observed to have a better energy dissipation ability, enhanced shape recovery, higher re-centering capability, and minor residual strains when compared with steel’s stress–strain curve. Furthermore, NiTi displayed a relatively considerable yield and ultimate strength under tensile testing.

### 3.2. Forms of NiTi

Like all SMA, NiTi also exists in two forms: superelastic and shape memory effect. The phase temperatures and crystal phase are two important parameters in deciding their behavior or form. The Austenite phase accounts for superelasticity, and the martensite phase for the shape memory effect. Table 1 displays a comprehensive study of published research papers on NiTi incorporation in structural applications as an embedded element in concrete. It is a summary of 70 NiTi-concrete-oriented research papers where the application, type of loading/test, form of NiTi used, research method, and findings/conclusion, for each paper have been briefly noted.

Superelastic NiTi can be effectively used for reinforcing structures in seismic zones due to its good self-centering ability, high strength, adequate energy dissipation, and superior hysteretic behavior over steel. Alternatively, the shape memory effect is mostly utilized in applications that depend on the recovery stress, such as prestressing, confinement, and strengthening [6,7,15,16]. However, as per Table 1, the number of papers that utilized the superelasticity feature of NiTi much more than those that utilized the shape memory effect of NiTi. A possible reason might be the ease of applicability of superelastic NiTi over the shape memory effect of NiTi. For instance, a superelastic NiTi bar does not require any means of stimulation when embedded in a beam to recover its shape other than unloading the beam, while a shape memory effect NiTi strip will require a heat source to recover its original shape. Superelastic NiTi exhibits excellent superelastic behavior owing to its high strain recoverability.

**Table 1:** Collected data from the published work on NiTi incorporation in concrete.

Ref.	Structural Application of NiTi	Test Type/Loading	Form of NiTi	Research Method	Conclusions
[6]	Concrete prestressed with NiTi	Tensile test 3-point bending test	wires (SME)	Experimental	Ability to attain prestressing
[7]	NiTi-confined concrete columns	uniaxial compressive loading eccentric uniaxial compressive loading	wires (SME)	Experimental	Ductile response controlling crack opening and propagation ability to deform significantly before failure
[11]	NiTi in cement mortars	pullout test	fibers (SME)	Experimental	increase in stiffness and ultimate strength of NiTi bars due to cold drawing increase in pullout resistance and bond strength due to cold drawing The enhancement of the bond strength by cold drawing and heat treatment was higher in mortar matrices of higher strength crack-closing potential in fiber-reinforced cement composites
[16]	Reinforced concrete beam strengthened temporarily by NiTi and permanently by CFRP plates	3-point bending test	wires (SME)	Experimental	Closure of cracks less deflection less residual deflection with higher fiber content
[17]	Self-compacted concrete reinforced with NiTi	Compressive strength test splitting tensile strength test flexural strength test impact test.	Half-circle hooked ends Fibers (SE)	Experimental	Better fresh properties Considerable compressive strength Considerable splitting tensile strength Higher peak fracture strength and peak strain Improved energy dissipation Sudden impact force resistance.
[18]	Concrete Beam reinforced by NiTi	3- and 4-point bending test	Bars (SE)	Numerical	Slightly higher elasticity Less residual deflection Slightly higher maximum stresses Decrease in residual stresses. Better recovery
[19]	Embedment of NiTi in cementitious slab	4-point bending test	crimped shape fibers (SME)	Experimental	Recovery of flexural displacement closure of cracks
[20]	concrete shear walls reinforced with NiTi	monotonic and cyclic loading.	bars (SE)	Numerical	Postponing of the strength degradation capacity Almost no residual deformations Peak strain is less than the maximum strain recovery limit Increase in ductility Increase in ultimate displacement
[21]	concrete shear walls reinforced with NiTi	Push-over and reverse cycling	bars (SE)	Numerical	Similar Lateral Strength Capacity with Steel Reinforced Wall Similar Lateral Displacement Capacity with Steel Reinforced Wall Higher Restoring Capacity
[22]	Concrete columns reinforced with NiTi	Seismic loading	bars (SE)	Numerical	Significant reduction in the maximum residual drift
[14]	Concrete beam reinforced with NiTi	monotonic loading cyclic loading reverse cyclic loading	bars (SE)	Experimental	limited residual displacements and crack widths Higher yield and ultimate loads comparable displacement ductility to conventional reinforced beams Comparable energy dissipation to a conventional reinforced beam under cyclic loading Less energy dissipation than a conventional reinforced beam under reverse cyclic loading
[23]	Concrete beam reinforced with NiTi	2-point symmetric loading	bars (SE)	Experimental	Residual displacement less than that of the steel-reinforced beam Full recovery of strains Lower stiffness of NiTi-reinforced beams Alternative members with High-strength steel and NiTi can provide reasonable stiffness and partial deformation recovery.
[24]	Retrofitting of beam-column joint by post-tensioned NiTi	quasi-static cyclic loading	bars (SE)	Experimental	enhanced ductility better energy dissipation higher load carrying capacity reduced cracking improved stiffness degradation
[25]	Concrete columns reinforced with NiTi	Ground motion using a shaking table with an axial load applied to the column	bars (SE)	Experimental	small residual displacements Improved energy dissipation capacity Ability to recover nearly all of post-yield deformation.
[26]	RC beams retrofitted with NiTi-ECC composite materials	Quasi-static cyclic test adopting 4-point bending loading	bars (SE)	Experimental	Less crack width fewer cracks better recovery performance good energy dissipation slightly less tensile capacity Lower bond strength
[27]	NiTi-reinforced bridge piers	Seismic loading	bars (SE)	numerical	better energy dissipation better re-centering performance better deformation capacity reduction in residual displacement reduction in residual drift ratio improved post-earthquake functionality
[28]	Beam-column joint reinforced with NiTi	reversed cyclic loading	bars (SE)	Experimental	the ability to recover most of the post-yield deformation Improved seismic performance ability to reduce damage Less energy dissipation
[29]	Concrete shear wall reinforced with NiTi	Axial Load	bars (SE)	Numerical	higher energy dissipation higher self-centering capacity Lower stiffness Higher yield drift ratio
[30]	Concrete beam reinforced with NiTi	Four-point bending load	bars (SE)	Numerical	stiffer beam with percentage of NiTi bars Higher recovery ratio with a higher percentage of NiTi bars Reduced residual displacement More NiTi bars with thinner diameters provide higher cracking load and less residual displacement than fewer bars with thicker diameters
[31]	Concrete beam reinforced with NiTi	3-point bending test under displacement control	cables (SE)	Experimental	good strain recovery Low modulus of elasticity acts as a limiting factor
[32]	Self-compacted concrete reinforced with NiTi	Slump flow and J-ring test 4-point static flexural test 4-point cyclic flexural test	half-circle hooked ends fibers (SE)	Experimental	Improvement in flexural strength Higher re-centering ability Tougher concrete Increase in peak load Increase in energy absorption postponing of initial cracks restriction of crack width
[33]	NiTi-FRP reinforced concrete frames	sequential ground motions	Bars (SE)	Numerical	improvement in seismic behavior reduction in residual drifts superior energy dissipation accumulation of lower residual drifts
[34]	RC beams strengthened with NiTi in combination with adhesive released from hollow fibers	Bending test	Wires (SE)	Experimental	addition of self-restoration capacity to concrete beams cracks closure Increase in cracking load Redistribution of stress to uncracked sections the ability to derive reserve strength from uncracked sections
[35]	fiber-reinforced geopolymer concrete incorporating NiTi, steel and polypropylene	compressive test splitting tensile test static flexural cyclic flexural tests	fibers (SE)	Experimental	moderate contribution to the mechanical properties of the concrete excellent enhancement in cracking resistance superior cyclic flexural performance with minimal residual deformation highest re-centering ratios in four cycles compared to SFRGPC and PPFRGPC mixes.
[36]	Concrete walls reinforced with NiTi	cyclic quasi-static tests	bars (SE)	Experimental	higher displacement ductility improvement in lateral residual displacement control Promising displacement recovery capacity reduction in residual vertical elongation
[37]	NiTi embedded in ECC	Flow table test Ultrasonic pulse velocity test Compression test Three-point bending test Uniaxial direct tension test	fibers (SE)	Experimental	reduction in residual crack width Improved cracking strength, mid-span displacement, and ultimate strain with the increase in NiTi fiber dosage Better fractal dimension and surface fracture energy dissipation as the NiTi fiber dosage increases. Robust tensile strain hardening behavior along with saturated multiple cracking features in hybrid fiber (NiTi + PVA) reinforced ECC
[38]	Active confinement to non-circular concrete elements using NiTi	Monotonic and cyclic uniaxial compression loads	wires (SME)	Experimental	significant improvement in ultimate strain increase in residual strength of concrete
[39]	Bridge piers incorporating ECC and NiTi	low-cycle horizontal reciprocating loading	bars (SE)	Experimental	improvement of deformation ability and ductility Less damage than conventional concrete specimens better self-centering effects excellent seismic performance
[40]	NiTi RC buildings	triangular lateral load	bars (SE)	Numerical	Ability to initiate major progress in seismic design higher inter-story and roof drift due to the low modulus of elasticity of NiTi
[41]	Concrete columns reinforced with NiTi and ECC	lateral cyclic loading	bars (SE)	Numerical	increase in load-carrying capacity higher displacement capacity higher rotational capacity higher drift ratio negligible or zero residual drift
[42]	Precast Column-to-foundation connection with NiTi reinforcement and UHPC in column	lateral reversed cyclic loading constant axial load	bars (SE)	Experimental	enhancement of deformation capacity and energy dissipation
[43]	NiTi in mortar beams	4-point bending test crack-closing tests	fibers (SE)	Experimental	flexural capacity improvement improvement in crack-closing capacity Higher fiber content leads to better crack-closing performance Ability to improve the durability and mechanical properties of cement or concrete structures.
[44]	NiTi in cement mortar beams	Three-point bending tests	straight fibers (SME) dog-bone fibers (SME)	Experimental	higher modulus of elasticity, yielding stress, and ultimate stress in NiTi fibers than in NiTiNb fibers Better ductility in NiTiNb fibers Diameter and length recovery of NiTi are more efficient than those of NiTiNb fibers Residual strength is greater for straight fibers than dog-bone fibers. The reduction ratio of the post-cracking flexural strength decreased as the number of fibers increased The crack closing ratio of the straight fibers was higher for the NiTi than the NiTiNb fibers Compared to the dog-bone NiTi fibers, the straight NiTi showed better crack-closing performance
[45]	Mortar reinforced with NiTi	direct tensile test	crimped fibers (SME)	Experimental	improvement of the tensile behavior of mortars by the passive action of the bond resistance and active action of the SME 1 mm fibers did not contribute to the increase in tensile strength as the 0.7 mm fibers did. Passive action contributed more than active action to increase tensile strength. The tensile strength increased significantly with the NiTi fiber volume fraction increase. Fiber volume fraction or recovery stress did not influence cracking strength.
[46]	NiTi RC beams	Four-point cyclic flexural test	double-hooked-end fibers (SE)	Experimental	Increasing the volumetric ratio of fibers and compressive strength of the matrix led to higher flexural strength and toughness, deflection recovery, and crack closing performance. HP concrete mix with 1% NiTi fibers showed the highest toughening and displacement recovery performance a fiber volume fraction between 0.75% and 1% in NS mixtures and at least 1% in HP composites can be adopted for better flexural resistance
[47]	Concrete shear wall reinforced with NiTi	cyclic lateral load with constant compressive load on top	bars (SE)	numerical and experimental	less peak lateral force higher drift capacity decreased yield load Less energy dissipation enhancement of residual lateral displacement enhancement of displacement capacity as the aspect ratio increased, the ductility factor decreased higher ductility factor in NiTi-reinforced walls
[48]	FRSCC beams reinforced with NiTi	pullout test tensile test 3-point bending test	fibers (SE)	Experimental	increase in flexural strength considerable flexural residual performance NiTi did not perform better than steel fibers due to the smooth surface of the NiTi fiber, non-hooked-end, and less designed fiber ratio.
[49]	NiTi in cementitious composite materials	pullout test	fibers (SME)	Experimental	efficiency of crimped NiTi fibers over other end-shape NiTi fibers
[50]	NiTi in self-repaired concrete beams	Four-point static bending cyclic load	bars (SE)	Experimental	increased ultimate load less deflection and strain strain recovery behavior
[51]	NiTi fiber Reinforced Concrete	compressive test	crimped fibers (SME)	Experimental	reduction in compressive strength under the passive action of the fibers compressive strength, elasticity modulus, and failure strain increased significantly as the fibers were heated to induce the prestressing effect. a significant rise in the composite’s toughness The increase in compressive strength is due to the crimped geometry of the NiTi fiber. The prestressing effect of this type of NiTi fiber geometry can lead to a noticeable improvement in the capacity of concrete to absorb energy
[52]	concrete shear walls reinforced with NiTi	ground motion	bars (SE)	numerical	Cu-based SMA RC wall exhibits superior performance regarding internal drift ratio, residual internal drift ratio, shear forces, bending moment, and responds well regarding material damage and collapse margin ratio compared to NiTi RC walls and FE-based SMA RC walls.
[53]	NiTi embedded in a concrete beam	Bending driven by embedded NiTi actuators	wires (SME)	Experimental	A large recovery force was obtained upon heating the NiTi wires NiTi wires could be used as actuators to change the deflection of a concrete beam
[54]	NiTi fiber reinforced concrete	Four-point bending test	hooked-end fibers (SE)	Experimental	Compared to steel fibers, RC, and NiTi FRC showed lower flexural strength but higher flexural toughness and deflection capacity. Compared to steel fibers, RC, and NiTi, FRC has slightly lower compressive strength and modulus of elasticity. NiTi FRC displayed better control over cracking, improved multiple cracking performance, and tighter crack profiles.
[55]	Prefabricated concrete frame joints with NiTi and ECC	Low-cycle reciprocating loading tests	bars (SE)	Experimental	reduction in residual deformation and improvement in self-centering capacity Cracks were restored after unloading ductility ability maintained at a high level
[56]	Concrete shear walls reinforced with NiTi	single curvature bending	bars (SE)	Experimental	reduction in permanent displacement and concrete damage Further research is required into seismic design parameters
[57]	Concrete shear walls reinforced with NiTi	cyclic lateral load	bars (SE)	experimental and numerical	Shorter plastic hinge length Slightly smaller inelastic rotational capacity
[58]	concrete beams reinforced with NiTi	semi-cyclic point loading	wires (SE)	Experimental	high ductility in shear very high deflection and large crack widths at failure ability to sustain a significant load after full development of the critical shear crack Enhancement of resisting mechanisms at failure More research is needed. NiTi spirals could be used for beam-column connections in different circumstances
[59]	hybrid NiTi/steel fiber reinforced concrete	Four-point cyclic bending test	fibers (SE)	Experimental	Due to their straight shape, adding NiTi fibers did not significantly improve the re-centering capabilities. excessive pullout preventing crack recovery or re-centering capability. better crack-width control NiTi fibers with end hooks could provide sufficient mechanical anchorage and induce flag-shaped super elastic response, leading to higher re-centering and crack closing characteristics.
[60]	RC frames enhanced by NiTi and UHPC	increasing peak ground acceleration	bars (SE)	numerical	the lower stiffness of NiTi, can significantly modify a building’s dynamic characteristics Designs with steel have lower drifts, but those with NiTi have lower residual drifts
[61]	NiTi-reinforced concrete frames	sequential ground motions	bars (SE)	numerical	Hybrid plastic hinge (steel + NiTi) frames have improved lateral shear capacity compared to NiTi-reinforced frames Negligible floor acceleration for both frame systems HBH frames have better energy dissipation and consequently lower inter-story drifts compared to NiTi-reinforced frames The same residual drift ratio is found for both systems, but with lower construction costs for HBH frames.
[62]	NiTi-confined RC columns	concentric uniaxial compressive loading	wires (SME)	numerical	The analytical model predictions match the experimental test data of externally NiTi-confined RC columns. For future research, the developed analytical model to predict the concentric response of SMA-confined RC columns can be utilized to predict the load (P)—moment (M) interaction response.
[63]	NiTi and steel-reinforced concrete shear walls	lateral cyclic loading	bars (SE)	experimental	NiTi-reinforced concrete components are self-centering and permit the repair of damaged areas. Ability to reuse NiTi bars for repair applications due to their shape memory effect. Repaired walls restored the yield and ultimate lateral load capacities but sustained lower drift capacities. The wall recovered some of the imposed lateral drift. The wall dissipated more energy.
[64]	Beam-column joint reinforced with NiTi	reverse cyclic loading	bars (SE)	numerical	improvement of seismic performance adequate energy dissipation capacity Lower residual deformations
[65]	RC columns with HPFRC and NiTi	constant axial load and cyclic lateral load	bars (SE)	experimental	high displacement ductility low residual drift minimal damage higher lateral strength greater energy dissipation
[66]	HPC and VHPC elements with NiTi reinforcements	constant axial load and cyclic lateral load	bars (SE)	experimental	higher maximum lateral load better-distributed crack patterns greater energy dissipation maximum lateral load decreased with tie spacing and shear slenderness and increased with longitudinal reinforcement ratio.
[67]	Concrete walls reinforced with NiTi	axial load varying bending moment reverse cyclic lateral load	bars (SE)	experimental	concentration of strain at the base of the wall nonlinear strain profiles across the section limitations in the Bernoulli-Euler assumption for RC walls with NiTi rebars. need for further research to investigate the behavior of RC walls with NiTi rebars under seismic events and the validity of certain assumptions in the design and analysis of such walls.
[68]	mortar reinforced NiTi	axial cyclic compressive test	crimped shape fibers dog-bone-shaped fibers	experimental	Increase in cyclic peak strength reduction in the plastic strain of FR mortar A higher NiTi fiber content resulted in a stronger positive effect on the peak strength and plastic strain. SME of NiTi fibers provided bridging capacity and recovery stress SME of NiTi fibers caused a delay in crack initiation and crack propagation
[69]	ductile fiber reinforced cement-based composite beams incorporating NiTi	Cyclic point load	Bars (SE)	experimental	Appealing self-centering properties very minor residual deformations
[70]	RC-MRFs with UHPSFRC and NiTi	axial load reversed cyclic loading	Bars (SE)	numerical	Usage of NiTi bars causes a decrease in residual drifts and an increase in transient drifts The usage of UHPSFRC and NiTi bars leads to a reduction in both residual and transient drifts. increase in post-earthquake functionality decrease in repair cost Experimental research is also needed to study the seismic behavior of frames with NiTi and UHPSFRC
[71]	NiTi in mortar beams	cyclic 3-point bending test	straight wire crimped wire dog bone wire anchor reinforced wire (SME)	experimental	efficient displacement recovery ratio. The ratio is almost the same for all forms of wires. Beams with crimped wires have the highest load-bearing capacity; then come beams with straight wires and dog bone wires equally, and finally, the beams with anchor-reinforced wires. Compared to superelastic NiTi bars, the SME ability of NiTi wires is proven to fulfill the re-centering capacity.
[72]	RC beams strengthened by NiTi	cyclic loading	Strands (SE)	experimental	High-strength concrete beams strengthened by Niti strands show more ability to recover from flexural cracks. The recovering capacity of midspan deflection and flexural crack width in SMA-NSC beams is more than that of their corresponding HSC beams. better distribution of cracks in both specimens. Higher energy absorption capacity and residual stiffness. more ductile behavior.
[73]	Self-compacted cementitious composites with NiTi	slump test four-point bending test compression test direct tensile test electrical conductivity test	fibers (SE)	experimental	The addition of NiTi fibers slightly reduced the relative slump An increase in NiTi content enhanced the flexural and tensile post-peak performance up to 1% of NiTi fiber content, compressive strength decreased, but increased with higher contents. Adding NiTi fiber did not affect the composite conductivity
[74]	NiTi embedded in cement mortar	pullout test	prismatic and straight end fibers L-shaped end fibers N-shaped end fibers crimped end with spearhead fibers (SE)	experimental	The crimped end with spearhead fibers has the highest pullout resistance, flag-shaped behavior. The N-shaped end fibers have the lowest pullout resistance. Increasing the cross-sectional area of the crimped fibers or decreasing the upper plateau could enhance their functionality.
[75]	NiTi in ultra-high ductility fiber reinforced cementitious composite	flowability test 3-point bending test compressive test direct tensile test.	fibers powder (SE)	experimental	Nitinol powder accelerates the hydration of cement and increases flexural strength and compressive strength. Nitinol fibers lost nearly half their ductile potency after 90 days of curing. Nitinol powder sustained its ductility throughout the curing duration. Nitinol powder showed potential as a ductile cementitious composite without using fibers.
[76]	NiTi in mortar	pullout test	crimped fibers (SME)	experimental and numerical	greater pullout stress with a larger diameter Reduction in peak stress with increasing slip is smaller for larger diameters Significant stresses at the free portion’s summit point decrease as the location is further from the summit point.
[77]	NiTi embedded in cementitious composites	pullout test	straight fiber single-bend fiber double-bend fiber triple-bend fiber. (SE)	experimental	Compared to straight steel, straight fibers have lower pull-out performance The bending-unbending mechanism was insufficient to increase the maximum pullout load of NiTi fibers to the phase transformation stress, meaning further bond enhancement is required. The triple-bend fiber improved the average pull-out load and slip at a given loading rate. The effect of enhancing mechanical interlocking from 3D to 5D hooks on the fiber-matrix bond strength was more pronounced in steel fibers than NiTi fibers.
[78]	concrete composites reinforced with steel and NiTi	workability test compressive test 4-point bending test	fibers (SME)	experimental	An optimum mix ratio with the inclusion of hybrid fibers was achieved The inclusion of fibers improved the ductility and controlled the deflections Strain recovery was achieved by heat treatment of NiTi fibers The maximum performance of the structural components can be achieved with minimum fiber volume The inclusion of NiTi fibers has contributed to improved ductility and strain recovery A hybrid fiber combination can be efficiently used to minimize the crack width upon heat treatment
[79]	NiTi and PVA reinforced ECC	tensile test impact test	fibers (SME)	experimental	NiTi fibers significantly enhanced the tensile and impact performance of the ECC. Adding fibers beyond a certain dosage led to fiber clustering; thus, no further gain in tensile and impact performance was measured The impact resistance of specimens was further improved after exposure to heat treatment
[80]	NiTi and PVA reinforced ECC	flow table test compressive test splitting tensile test 4-point bending test	fibers (SME)	experimental	A combination of PVA and NiTi fibers significantly enhanced the tensile and flexural capacity of the ECC No further improvement in mechanical behavior was achieved beyond a certain fiber dosage due to increased porosity and fiber clustering. Despite the damage incurred by coexisting PVA fibers due to heat treatment, cracked SMA-ECC specimens were self-healed upon heat treatment owing to the self-centering capability of NiTi fibers.
[81]	NiTi in ECC	uniaxial cyclic tensile test	fibers (SE)	experimental	The addition of NiTi fibers improves the ultimate strain and ultimate tensile strength of ECC Increasing fiber content effectively enhances the strain and crack recovery rates Both too large and too small SMA fiber diameters lead to reduced recovery rates. 0.5 mm fibers led to the highest strain and crack recovery rates.
[82]	NiTi and CFRP sheets to strengthen concrete walls	Lateral cyclic loading	Sheets (SE)	Experimental	A remarkable increase in lateral load capacity An enhancement in energy dissipation.
[83]	NiTi bars for concrete frames	Static pushover analysis Nonlinear time-history dynamic analysis	Bars (SE)	Numerical	Improvement in seismic resistance Better re-centering performance
[84]	NiTi cables grid reinforced ECC	Flexural testing of reinforced concrete beams	Cables grid (SE)	Experimental and numerical	Improves flexural performance compared to unstrengthened RC beams Enhances bearing capacity and self-centering ability

### 3.3. Methodology of Research

The analysis of research methods used in studies on NiTi Shape Memory Alloys (SMA) for civil engineering applications (i.e., incorporation in concrete or cement-based composites) reveals that the majority relied on an experimental approach. This strong preference underscores the importance of physical testing to directly observe the performance of NiTi SMA under various structural conditions. Meanwhile, other studies employed a numerical approach, utilizing computational models to simulate and predict structural responses, often in cases where experimental setups are limited or require parametric exploration. A much smaller proportion of studies combined both methods, demonstrating an integrated framework for validation and prediction.

## 4. Applications of Nitinol

Despite NiTi’s first incorporation into civil engineering applications in the 90s, it was not frequently used in the field until recently. Over the last decade, researchers’ interest in integrating NiTi in structural applications has grown. Different structural applications of NiTi in concrete have been published in the past years. NiTi SMA was applied in self-compacted concrete reinforced with NiTi, demonstrating its functional benefits in civil engineering applications [17,32]. It was also used in concrete beams reinforced by NiTi, improving flexural performance and energy dissipation [18] and in embedment within cementitious slabs for adaptive stress redistribution and damage control [19]. It has been utilized in reinforced concrete beams strengthened temporarily by NiTi and permanently by carbon fiber reinforced polymer (CFRP) plates, enabling reversible retrofitting [16]. Meanwhile, concrete shear walls have been reinforced with NiTi to enhance seismic resilience and re-centering capacity [20,21,52,56,57,83]. In concrete columns, NiTi reinforcement and confinement have contributed to improved ductility and post-yield behavior [22,25]. Applications in prestrained or anchored NiTi bars in beams have demonstrated effective prestressing and strengthening functions [14,36,40]. NiTi wire jackets have been used extensively to retrofit columns, frames, and plastic hinge regions, improving deformation capacity and earthquake resistance [23,24,26,27,28,30,83]. NiTi SMA wires and cables have also been embedded internally or applied externally in RC beams to improve load distribution, energy dissipation, and crack control [29,31,34,35,37,38,39,41,84]. Strengthening of prestressed or damaged girders and beams using NiTi and CFRP has shown promising results [33,34]. NiTi SMA has been incorporated into slabs and frames to enable self-centering and intelligent response to loading [42,43]. In seismic applications, NiTi SMA dampers and superelastic devices have been employed to retrofit joints, piers, and frames, enabling energy dissipation and damage mitigation [44,45,47,48,49,50,63,66,70]. NiTi spirals and reinforcement have been used in columns and base isolation systems for enhanced ductility and cyclic performance [46,58,59,61]. Furthermore, NiTi has been implemented in bridges and isolation systems through energy-dissipating bearings, sliding supports, and spiral springs [51,53,54,55,60,62,64]. Smart concrete elements incorporating NiTi have enabled active performance control and damage recovery [67,68,69,71].

**Figure 8:**
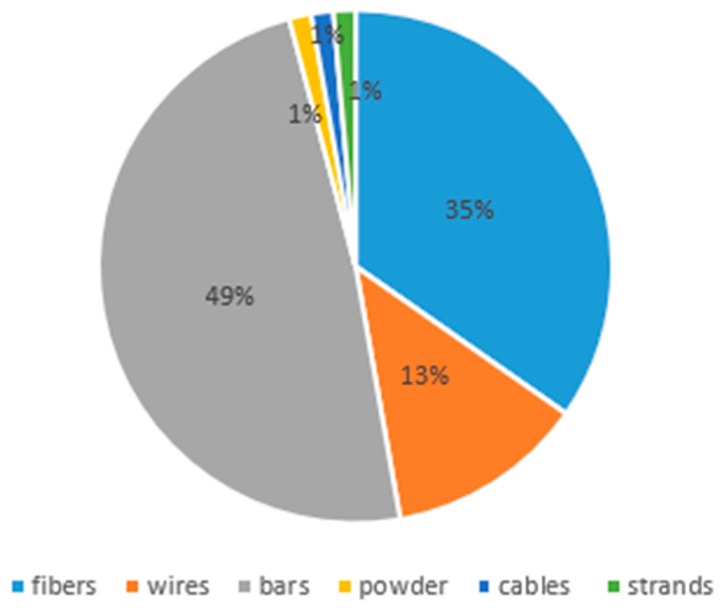
NiTi forms and their usage frequency in structural applications based on the collected data.

Figure 8 demonstrates the percentage distribution of different kinds of NiTi in civil engineering applications based on the collected data, including bars, fibers, wires, cables, strands, and powder. Since more than 95% of the studies examined herein aimed to integrate NiTi SMA in the first three forms of NiTi (i.e., bars, fibers, and wires), the focus of this review was on said forms of NiTi. Mas et al. [31] placed a NiTi cable into a real beam specimen to work as a longitudinal reinforcement and tested it under a 3-point bending test. Their results showed good strain recovery, which presented an opportunity for NiTi to be used as a reinforcer, considering NiTi’s low modulus of elasticity, which acts as a limiting factor and must be improved. Azadpour and Maghsoudi [72] used NiTi as strands in their experiments to strengthen continuous RC beams and test them under cyclic loading. Two types of concrete were used for beam specimens: normal-strength concrete (NSC) and high-strength concrete (HSC). The results displayed better distribution of cracks, higher energy absorption capacity and residual stiffness, and more ductile behavior for both HSC and NSC beams. Nevertheless, HSC beams strengthened with NiTi strands demonstrated a higher ability to recover from flexural cracks, whereas the recovery capacity of midspan and flexural crack width in NSC beams, strengthened by NiTi strands, exceeded that of their corresponding HSC beams [72].

In one study, Gideon and Milan [75] investigated the effect of NiTi fibers and powder on ultra-high ductility fiber-reinforced cementitious composite. The NiTi fibers and powder were utilized as micro-reinforcement and reactive agents, respectively. Test outcomes revealed that NiTi powder expedites cement hydration and enhances TiO_2_ nucleation onto C-S-H, augmenting flexural and compressive strength. The cementitious composite incorporating NiTi fibers achieved ductility of 7.3% and 7.8% after 28 days of curing. However, these composites could not sustain their ductility after 90 days, experiencing nearly a fifty percent reduction. Conversely, NiTi powder exhibited 2.8% and 5.6% ductility after 28 and 90 days of curing, respectively, suggesting its potential as a ductile cementitious composite in the absence of fibers.

### 4.1. NiTi Bars

**Figure 9:**
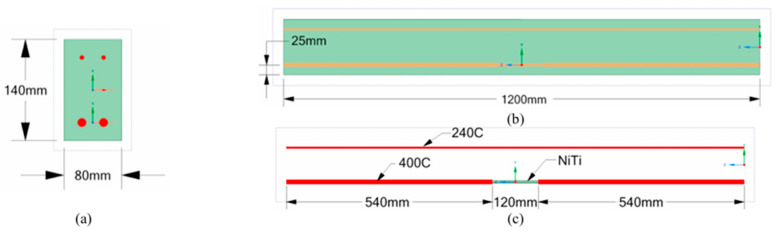
Beam model with NiTi reinforcement: (**a**) cross-section; (**b**) right side; (**c**) reinforcement [18].

The use of NiTi bars in civil engineering applications is rising due to their unique properties and potential benefits in enhancing the performance and durability of concrete structures. These properties include, but are not limited to, high strength-to-weight ratio, corrosion resistance, and excellent fatigue resistance. These properties make NiTi bars suitable for seismic retrofitting, structural intervention of historical structures, vibration mitigation, and reinforcement of concrete beams, walls, columns, joints, and frames. With reference to Figure 8 and Table 1, bars are the most used NiTi form in civil engineering applications. Several papers have reported the utilization of NiTi bars in beams as reinforcers [14,18,23,26,30,50,69]. One form of NiTi reinforcement is presented in Figure 9. Bykiv et al. [18] performed linear structural analysis using finite element modeling (FEM) to study the behavior of reinforced concrete beams with NiTi and steel. Only the middle portion of the bottom reinforcement was NiTi bars. The specimens were tested by simulating 3 and 4-point bending tests. Similarly, Abdulridha et al. [14] exploited NiTi bars only in the middle portion of the reinforcement. Their research aimed to explore the structural behavior of superelastic NiTi-reinforced concrete and to develop an initial constitutive model applicable to nonlinear finite element simulations. Seven flexure-critical concrete beams, supported simply, were used for the study. These beams were reinforced with either SMA bars in the critical zone or conditional deformed reinforcement. They underwent various loading conditions, including monotonic, cyclic, and reverse cyclic loading.

Saiidi et al. [23] investigated small-scale concrete beams reinforced with NiTi under half-cycle loads, while Qian et al. [26] tested four beam specimens by enlarging them with a concrete section. Each specimen was enlarged using a different kind of enlarging section. One specimen was enlarged with a steel-reinforced section, another with a NiTi-reinforced section, the third with a steel-reinforced engineered cementitious composites (ECC), and the last with NiTi-reinforced ECC. These specimens were tested under a quasi-static cyclic test adopting 4-point bending loading. Molod et al. [30] numerically investigated the role of diameter and percentage of NiTi as the main reinforcement of concrete beams in increasing stiffness and reducing residual displacement. To do so, eight beams with the same geometry and boundary conditions reinforced with superelastic NiTi have been modeled in Ansys APDL and tested under two cycles of four-point bending loading. Hassan et al. [50] introduced an experimental initiative that examined the impact of employing NiTi bars alongside internal injection techniques to repair and strengthen cracked reinforced concrete beams.

Hung and Yen [69] explored using NiTi bars in reinforced concrete (RC) beams for enhanced seismic performance. Four beam specimens with experimental parameters, including ductile fiber-reinforced cement-based composites (DFRCCs), NiTi bars, and bond strength between rebar and DFRCCs, were designed and tested under cyclic point loading. Overall, it is concluded that NiTi-reinforced beams present several benefits over conventional steel reinforcement. They demonstrate slightly higher maximum stresses and elasticity and decreased residual deflection and stresses, leading to a better recovery performance. NiTi reinforcement also results in limited and controlled crack widths and residual displacements, enhanced yield and ultimate loads, and comparable displacement ductility to traditional reinforced beams. Additionally, NiTi bars enable full recovery of strains and demonstrate good energy dissipation characteristics. Anyhow, NiTi-reinforced beams possess slightly lower tensile capacity and less stiffness when compared to steel-reinforced beams. As the quantity of NiTi bars increases in a beam, the beams show increased stiffness, cracking load resistance, and reduced residual displacement, making them suitable for applications requiring high-strength and partial deformation recovery. Eventually, NiTi reinforcement offered appealing self-centering properties and minimal residual deformations [14,18,23,26,30,50,69]. NiTi bars were also used as a reinforcement in RC walls [20,21,29,36,47,52,56,57,63,67].

Abraik and Asteetah [20] proposed and studied a slotted RC wall reinforced with superelastic NiTi bars. The seismic performance of the NiTi-slotted RC wall was numerically investigated using various parameters of interest in seismic applications, and it was evaluated through monotonic and cyclic loading and compared to conventional and high-performance RC walls. A study by Soares et al. [21] investigated the performance of hybrid NiTi-steel reinforced concrete. Nonlinear finite element modeling was employed to analyze the response of these hybrid walls under push-over and reverse cyclic loading. Kian and Noguez [29] conducted three experiments incorporating NiTi bars. In the first study, they focused on creating validated analysis models through advanced RC finite element analysis software called VecTor2. A parametric investigation was conducted on three variations in self-centering shear walls, each differing in aspect ratios, axial load ratios, and reinforcement ratios. The three versions of shear walls were reinforced with NiTi bars, glass fiber reinforced polymer (GFRP) bars, or post-tensioned high-strength steel strands reinforced walls. Subsequently, the paper explained how the data could be utilized to establish design guidelines for the examined shear walls. Their second study investigated the performance of three walls made with fiber-reinforced composites and reinforced with steel rebars, incorporating self-centering reinforcements like NiTi bars, GFRP bars, or high-strength steel strands. These walls were compared to a conventional RC shear wall under single curvature bending. The analysis investigated parameters like inelastic rotational capacity, plastic hinge length, and self-centering, assessing their alignment with North American seismic design code [56].

In one research, a parametric study was introduced on the plastic hinge length and inelastic rotational capacity of three types of self-centering RC shear walls under cyclic lateral loads. The walls were reinforced with conventional steel and NiTi bars, GFRP bars, or post-tensioned high-strength steel strands [58]. Almeida et al. [36] explored the utilization of shape-memory NiTi alloy rebars as substitutes for steel in reinforced concrete walls. The study involved cyclic quasi-static experimental tests on flexural controlled two large-scale units. Dina et al. [47] conducted an experimental and numerical study on large-scale RC shear walls reinforced with NiTi bars. Specifically, they aimed to study the effect of the intensity of NiTi on the cyclic response. In the test setup, the walls were subjected to cyclic lateral load with a constant compressive load applied on the top. Abraik and Assaf [52] investigated the impact of ground motion duration on self-centering (RC) shear walls reinforced with superelastic NiTi. Using a ten-story building model, seismic performance metrics such as inter-story drift ratios, shear forces, and bending moments were analyzed under varying ground motion durations. Puentes et al. [63] conducted reverse cyclic load testing on repaired slender concrete shear walls, comparing specimens reinforced internally with superelastic NiTi to walls repaired using deformed mild steel reinforcement. Hoult and Almeida’s [67] paper explored the potential of reducing residual displacements in reinforced concrete walls by replacing local segments of reinforcing steel with NiTi.

The conclusion drawn from these papers is that adding NiTi to concrete walls led to postponement of strength degradation, reduced residual deformations, and higher ductility and ultimate displacement. The NiTi-reinforced walls demonstrated similar lateral strength and displacement capacities as steel-reinforced walls but experienced higher recovery capability, energy dissipation, and self-centering ability. However, they have lower stiffness. NiTi reinforcement improves residual displacement control and recovery and reduces concrete damage. Despite being promising, further research is required to refine seismic design parameters and address limitations [20,21,29,36,47,52,56,57,63,67,82].

Besides RC beams and walls, NiTi is also used in RC columns [22,25,27,39,41,65,66]. Lee et al. [22] conducted a study that focused on deriving an expression for the plastic hinge length of a rectangular concrete column reinforced with NiTi. Saiidi and Wang [25] studied the usage of NiTi bars in the plastic hinge of concrete columns in hopes of reducing residual displacement. In addition, they evaluated the seismic performance and damage of NiTi-reinforced columns that were repaired using ECC. Rahman and Muntasir Billah [27] numerically examined the influence of NiTi bars on the seismic behavior of bridge bents when exposed to long durations of motion. They compared NiTi-reinforced bent and conventional steel-reinforced bent.

Qian et al. [39] compared five types of bridge pier specimens: ordinary reinforced concrete, reinforced ECC, steel strand reinforced concrete, steel strand reinforced ECC, and SMA bar ECC. Low-cycle repeated loading tests were conducted to evaluate seismic performance regarding failure mode, bearing capacity, ductility, and energy dissipation capacity. George et al. [41] also used ECC and NiTi in their experiments. Their study numerically evaluated the seismic performance of RC columns with NiTi in the plastic hinge region under reverse cyclic loading with a constant axial load on the top. Eventually, this specimen was compared with conventional steel-reinforced and steel-reinforced columns with ECC in the critical region. With a different approach, Barcelo et al. researched hybrid-reinforced concrete columns containing NiTi and High-Performance Fiber Reinforced Concrete (HPFRC) in the critical end regions. The specimens’ behavior was assessed under cyclic loading [65]. Finally, and out of the ordinary, Barcelo et al. [66] conducted an experimental study on the behavior of high-performance concrete (HPC) and very high-performance concrete (VHPC) columns with NiTi reinforcements in critical regions subjected to constant axial and lateral cyclic load combinations. As a conclusion of the previously reported papers, utilizing NiTi in columns resulted in superior seismic performance with a notable decrease in the maximum residual drift, resulting in minimal residual displacements. The specimens demonstrated better energy dissipation, deformation ability, and re-centering performance. Furthermore, they presented higher load-carrying capacity, ductility, lateral strength, minimal damage, and negligible residual drift, ensuring post-earthquake functionality.

**Figure 10:**
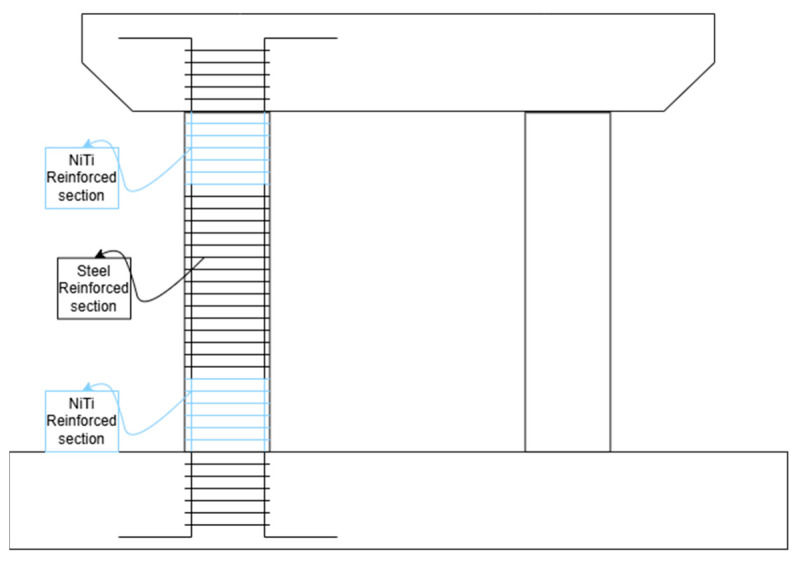
Bridge Bent elevation and reinforcement arrangement [28].

Several papers have reported the utilization of NiTi in beam-column and column-foundation joints [24,28,42,55,64], as shown, for example, in Figure 10. Qian et al. [55], Nahar et al. [64], and Youssef et al. [28] similarly exploited NiTi bars at the beam-column joint and investigated the specimens’ seismic performance under low-cycle reciprocating loading test and reverse cyclic loading, respectively. Yurdakul et al. [24] retrofitted non-seismically designed beam-column joints with post-tensioned NiTi and evaluated the influence of NiTi on their behavior under quasi-static cyclic loading. Bracelo et al. [42] studied a different type of joint: the column-foundation joint. They added NiTi reinforcement at the column-foundation joint of precast columns made with ultra-high-performance concrete to analyze their response experimentally under earthquake load. It has been observed that joints reinforced with NiTi exhibited enhanced ductility, better energy dissipation, higher load-carrying capacity, reduced cracking, and improved stiffness degradation. These properties play a role in improving seismic performance, reducing damage, decreasing residual deformations, and enhancing self-centering capacity in structures subjected to dynamic loads, making them promising candidates for applications requiring resilient and durable structural systems like earthquake zones.

In other work, NiTi has been used to reinforce structural frames [33,40,60,61,70]. Zafar and Andrawes [33] focused on enhancing the seismic performance of concrete moment-resistant frames (MRF) using a new type of reinforcement combining fiber-reinforced polymer (FRP) with embedded superelastic NiTi fibers. This method was implemented numerically, and the model was subjected to sequential ground motions. The results obtained were compared to those of conventional steel-reinforced MRFs. The result demonstrated that the proposed reinforcement performed better than conventional steel reinforcement as it enhanced the resilience of RC moment frames by reducing damage accumulation and maintaining lower residual drifts, thereby improving the overall post-earthquake functionality of structures. The other papers that investigated reinforcing concrete frames with NiTi tested the specimens under loads such as 13 far-field earthquake records, triangular lateral load, increasing peak ground acceleration, sequential ground motions, and reversed cyclic loading [40,60,61,70].

Results indicated that NiTi-based frames exhibit a lower probability of damage and collapse when subjected to seismic loading. Combining steel and NiTi demonstrated improved lateral shear capacity compared to NiTi-reinforced frames alone. Structures incorporating NiTi bars experienced reduced residual drifts and increased transient drifts. This reduction not only strengthened post-earthquake functionality but also decreased repair costs. Finally, it has been noted that further experimental research is required to fully comprehend NiTi’s seismic behavior under various configurations.

By analyzing these studies, NiTi-reinforced concrete structures have some key limitations. For instance, it has been reported that NiTi-reinforced beams have slightly lower tensile capacity and stiffness in comparison to steel-reinforced beams. NiTi-reinforced walls have lower stiffness compared to steel-reinforced counterparts, but they demonstrate high recovery capability, energy dissipation, and self-centering ability. The literature has also provided contradictory statements on the elasticity and stiffness of NiTi-reinforced beams, which might warrant further clarification. In this context, further research is needed to refine seismic design parameters and address limitations for NiTi-reinforced walls.

### 4.2. NiTi Fibers

**Figure 11:**
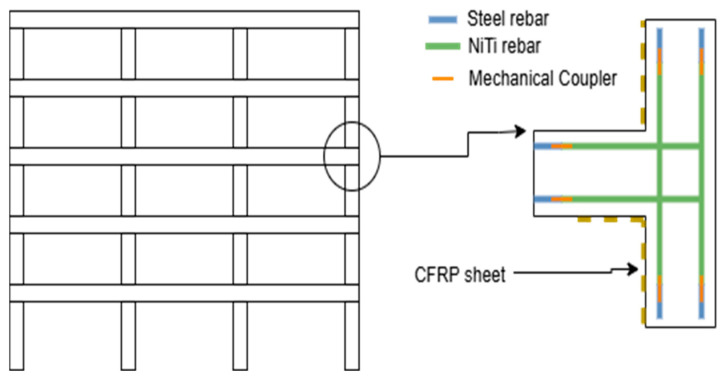
Model of RC frame with NiTi reinforcement and strengthened using CFRP sheets [8].

**Figure 12:**
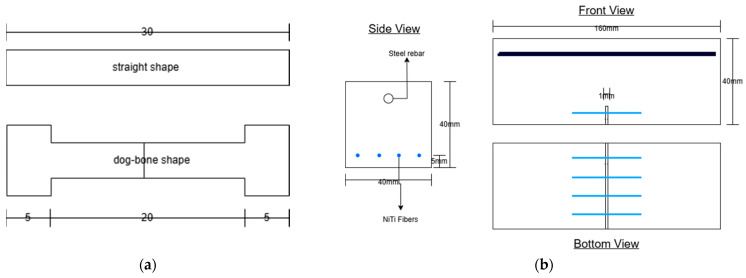
Geometry and dimensions of (**a**) NiTi fibers and (**b**) beam specimen [44].

Using different types of fibers, such as natural fibers, metallic, and polymeric fibers, to reinforce or overcome the brittleness of a specific material has been used for many years. NiTi can also be produced as fibers to reinforce concrete, mortars, and cementitious composites. By surveying the literature, the most common utilization area of fibers is mortars [11,43,44,45,68,74,76]. One application of fibers as a fiber-reinforced matrix is shown in Figure 11. Choi et al. [68] tested the compressive cyclic behavior of mortars reinforced with crimped and dog-bone-shaped NiTi fibers. In another study [74], they evaluated the tensile behavior of mortars with cold-drawn crimped NiTi fibers, considering the effects of fiber diameter, content, and recovery stress induced by heating. Finally, they investigated the pullout resistance of different shapes of fibers embedded in mortars under hysteretic pull-out testing. The shapes considered in their experiment were prismatic and straight-end fibers, L-shaped end fibers, N-shaped end fibers, and crimped ends with spearhead fibers. Similarly, Kim et al. [11] also investigated the pull-out resistance of NiTi fibers in cement fibers. Still, only straight fibers were considered, and treatments like cold drawing and heat treatment were utilized to enhance the pull-out performance of the fibers. Lee et al. [43] assessed the flexural capacity of cement mortar beams embedded with NiTi fibers. A four-point bending test was used with different fiber volume fractions. Ho et al. [76] studied the stress distribution of crimped NiTi fibers under pull-out force. Experimental tests and a finite element model were used for analysis. Finally, Lee et al. [44] evaluated and compared the crack closing capacity of NiTi and NiTiNb fibers in cement mortar beams utilizing their shape memory effect. Different geometries of fibers, including dog bone and straight, were used in previous work, as shown in Figure 12.

Results indicated that an increase in NiTi fiber content led to the enhancement of both flexural and crack-closing capacity in cement and concrete structures, improving overall mechanical properties and durability. NiTi fibers’ properties were dominant over those of NiTiNb fibers, such that NiTi fibers’ properties demonstrated better crack-closing ratios and tensile behavior, with smaller diameter fibers contributing significantly to improvement in tensile strength. In addition, cold drawing of NiTi boosted stiffness, pullout resistance, and bond strength. NiTi fiber’s shape memory effect was critical in enhancing cyclic peak strength and delaying crack initiation and propagation. Furthermore, the shape of fiber ends, such as crimped spearheads or flag-shaped fibers, significantly affected the pullout resistance, demonstrating the significance of fiber geometry in reinforcing cementitious materials. NiTi fibers’ applications were not limited to mortars only. Researchers also investigated the embedment of NiTi fibers in ECC [37,79,80,81].

In two different papers, Ali et al. conducted experiments on ECC reinforced with both PVA and NiTi, which they called hybrid-fiber reinforced ECC. Tests were conducted on the hybrid-fiber-reinforced ECC to report the effects of NiTi and PVA fibers on the ECC. Effects on parameters such as tensile performance, impact resistance, crack-healing, flexural capacity, and self-healing were analyzed [79,80]. Yang et al. [81] incorporated NiTi fibers in ECC and tested them under uniaxial tensile cyclic loading to understand the self-recovery of NiTi-reinforced ECC. Gurbuz et al. [37] also performed a case study on NiTi fibers in ECC. The case study consisted of an analysis of the mechanical properties of NiTi-reinforced ECC. Papers related to NiTi-reinforced ECC concluded that adding NiTi fibers to ECC results in notable improvements. These improvements included decreased residual crack width, better-cracking strength, mid-span displacement, and ultimate strain with increased NiTi fiber dosage. Increasing NiTi fiber dosage is associated with better fractal dimension and crack energy dissipation. Adding NiTi with PVA fibers further enhanced ECC’s flexural and tensile performance. Anyhow, there is a dosage limit beyond which mechanical properties are not improved due to fiber clustering and increased void spaces. Interestingly, it has been observed that heat treatment boosted impact resistance, and regardless of the damage to coexisting PVA fibers, cracked NiTi-ECC specimens underwent self-healing upon heat treatment, attributed to the shape memory effect of NiTi fibers. Eventually, optimizing the diameter of NiTi fibers was critical. Excessively large and small diameters decreased strain and crack recovery rates, while 0.5 mm fibers demonstrated the highest recovery rates.

Moving forward with the applications of NiTi fibers, cementitious composites are another category in which NiTi fibers have been embedded and studied [19,49,75,77,78]. Lee et al. [19] worked on developing a method for repairing and retrofitting concrete structures. NiTi crimped fibers with different volumes were embedded in a cementitious slab specimen and assessed under a 4-point bending test. The study concluded that using NiTi crimped fibers for repairing and retrofitting was feasible and led to the recovery of flexural displacement and closure of cracks. Choi et al. [49] assessed the bond strength, pull-out resistance, and crack-closing ability of crimped NiTi fibers produced from cold-drawn wires and embedded in cementitious composites. The crimped fibers were compared with different end shapes: straight and paddled. Eventually, the crimped fiber outperformed other end-shape fibers. Gideon and Milan [75] studied the impact of NiTi fibers on the ductility of ultra-high ductility fiber-reinforced cementitious composites. The effects of NiTi on the composite were concluded by conducting flowability, compressive, flexural, tensile, X-ray diffraction, and scanning electron microscopy (SEM) tests on the specimens. A part of their conclusion has highlighted that the NiTi fibers lost half of their ductility.

Dehghani and Aslani [77] embedded NiTi fibers with different hooked-end configurations in a highly flowable cementitious matrix. The paper focused on the influence of the different configurations and loading rates on the pull-out behavior of NiTi fibers embedded in cementitious composites. As a result, straight NiTi fibers demonstrated lower pull-out performance compared to straight steel alternatives. Additionally, the bending-unbending mechanism failed to increase NiTi fibers’ maximum pull-out load to their phase transformation stress, highlighting the need for further bond improvement. Conversely, 3D (triple bent) fibers showed improvements in the average pull-out load and slip at a given rate. Furthermore, improving mechanical interlocking from 3D to 5D hooks had a more notable impact on the fiber-matrix bond strength in steel fibers than NiTi fibers. Geetha and Selvakumar [78] studied concrete composites reinforced with steel and NiTi fibers regarding mechanical properties and ductility. Due to the positive influence of the fibers, they have referred to the composite as “a composite for the future”. It has been concluded that an optimal mix ratio incorporating hybrid fibers achieved enhanced ductility and controlled deflections. Heat treatment of NiTi fibers promoted strain recovery, contributing to the improved performance of structural elements with minimal fiber volume. Finally, incorporating NiTi fibers enhanced ductility and further assisted strain recovery. The hybrid fiber combination effectively reduced crack widths, especially when subjected to heat treatment.

It has also been recorded that NiTi fibers have been used in reinforced concrete [46,51,54,59]. Menna et al. [46] studied the flexural performance of hooked-end NiTi fibers reinforced concrete beams. Some specimens used in the experiment consisted of a normal concrete mix, and others consisted of a high-performance concrete mix. All specimens were reinforced with various volume fractions of NiTi fibers and subjected to a cyclic four-point bending test. The results showed that with a higher fiber volume ratio and higher matrix strength, flexural performance, deflection recovery, and crack closing ability increased. Choi et al. [51] investigated the effect of recovery stress using randomly distributed NiTi fibers in concrete. Cold-drawn crimped NiTi fibers were chosen for their ability to induce prestressing and provide strong bond resistance. Different aspect ratios of NiTi fibers were tested at the same volumetric fraction in reinforced concrete cylinders. The specimens were subjected to compressive strength tests. Results indicated improved ductility, increased compressive strength, and enhanced energy absorption capacity due to the NiTi fibers’ geometry in concrete. Dehghani and Aslani [54] discussed the experimental findings on concrete reinforced with NiTi fibers. Specimens with two types of end hooks were tested at varying fiber contents by volume (0.5% to 1.25%). Compared to steel fiber reinforced concrete (SFRC), NiTi fiber-reinforced concrete (NiTi FRC) exhibited lower flexural strength but higher toughness and deflection capacity. NiTi FRC also showed slightly lower compressive strength and modulus of elasticity than SFRC. NiTi FRC type 2, with 45° end hooks, was more effective in distributing stress and controlling cracking in the matrix. Meanwhile, Sherif et al. [59] explored using hybrid steel/NiTi fibers in RC beams under cyclic flexural loadings. Digital image correlation (DIC) tracked displacements and strains across the specimens. It has been concluded that the hybrid specimens exhibited reduced deflections at midspan and lower crack widths. On the other hand, adding NiTi fibers did not significantly improve the re-centering capabilities due to their straight shape, and excessive pullout prevented crack recovery or re-centering capability was observed. Consequently, NiTi fibers with end hooks could provide sufficient pullout resistance and induce a flag-shaped superelastic response, leading to higher re-centering and crack closing characteristics.

Furthermore, NiTi fibers have been incorporated not only in normal concrete mixes but also in different mixes, such as self-compacted concrete (SCC). Aslani et al. [17] conducted three studies incorporating NiTi fibers in SCC (NiTi FRSCC) and compared them to polypropylene fibers reinforced SCC and steel fibers reinforced SCC. They investigated and compared the fresh and mechanical properties, flexural and toughness properties, and tensile and bond characteristics of the different specimens. NiTi FRSCC displayed superior fresh properties and ductile performance compared to the polypropylene (PP) FRSCC and steel FRSCC. This was because NiTi fibers presented high ultimate tensile strength and superelasticity. These features delayed crack initiation and reduced crack widths in concrete, enhancing the ductility of the matrix and improving resistance to dynamic impacts [17]. Additionally, the shape memory alloy fiber-reinforced self-compacting concrete with a specific volume fraction (0.75%) presented the highest flexural strength, re-centering ability, and toughness compared to polypropylene and steel FRSCC. The experimental results indicated the advantage of NiTi’s shape memory and superelastic properties in delaying initial crack formation and constraining the crack widths [32]. Furthermore, it was found that the flexural strength of NiTi FRSCC increased, and the sample showed a considerable flexural residual performance without any fiber deformation or rupture. The steel and PPFRSCC samples also presented increased flexural strength with higher fiber volume fraction, but the steel FRSCC had the largest values due to its higher fiber tensile strength. It is essential to highlight that the NiTi sample did not exhibit better results than the steel sample due to the smooth surface of NiTi fibers, non-hooked-end, and less designed fiber ratio [48].

Finally, Table 1 recorded the utilization of NiTi fibers in self-compacted cementitious composites [73] and geopolymer concrete [35]. Dehghani and Aslani [73] explored the incorporation of NiTi in self-compacted cementitious composites regarding fresh, mechanical, and electrical properties. Outcomes were compared with those of steel fibers reinforced cementitious composites and carbon fibers reinforced cementitious composites. The addition of NiTi and steel fibers induced a slight decrease in the slump, while carbon fibers significantly reduced the flowability of the mixture. The flexural and tensile performance post-peak increased with higher NiTi and steel fiber content, especially at 1% to 1.5% by volume. Compressive strength initially dropped with up to 1% NiTi and steel fibers but increased with higher content. The conductivity of the composite was slightly affected by NiTi and steel fibers up to 1.5% by volume, while even the low content of carbon fibers (0.1%) significantly increased conductivity. Moreover, Wang et al. [35] investigated the mechanical properties of geopolymer concrete reinforced with NiTi, steel, and polypropylene fibers. NiTi, steel, and Polypropylene (PP) fibers were added to the geopolymer concrete (GPC) with varying fiber volume contents. Parameters like workability, compressive strength, splitting tensile strength, modulus of elasticity, static flexural strength, and cyclic flexural strength were tested to investigate the performance of steel fiber-reinforced GPC (SFRGPC), NiTi fiber-reinforced GPC (NiTi-FRGPC) and PP fiber-reinforced GPC (PPFRGPC). Results indicated that increasing steel and NiTi fibers enhanced the mechanical performance of FRGPC, while adding more PP fiber content reduced it. SFRGPC mixes presented the highest compressive, splitting tensile, and flexural strengths. NiTi-SMAFRGPC mixes showed outstanding cyclic flexural performance with minimal residual deformation and the highest re-centering ratios throughout four cycles compared to SFRGPC and PPFRGPC mixes.

The existing literature on NiTi fibers in cementitious materials highlights several limitations and areas needing further investigation. Specifically, in studies investigating pull-out resistance, the focus was exclusively on straight fibers, suggesting a gap in the understanding of other fiber geometries in their specific work. Moreover, straight NiTi fibers have demonstrated lower pull-out performance compared to straight steel counterparts. Additionally, the bending-unbending mechanism was insufficient in increasing the fiber maximum pull-out load to their phase transformation stress, highlighting a need for further bond improvement. This limitation extends to re-centering capabilities, where adding straight NiTi fibers did not significantly improve these properties, with excessive pullout preventing crack recovery.

When comparing NiTi fiber-reinforced concrete with steel fiber-reinforced equivalents, NiTi-based concrete exhibited lower flexural strength, compressive strength, and modulus of elasticity than steel-based counterparts. Similarly, in self-compacted concrete (SCC), NiTi fiber-reinforced SCC did not show better flexural strength than steel equivalents. This was primarily attributed to the smooth surface, non-hooked-end, and a lower fiber ratio of NiTi fibers, owing to fiber clustering and increased void spaces. Lastly, the effectiveness of NiTi fibers was also sensitive to their diameter, where excessively large and small diameters decreased the strain and crack recovery rates. Accordingly, precise optimization of fiber dimensions was noted to be critical for achieving maximum benefit.

### 4.3. NiTi Wires

**Figure 13:**
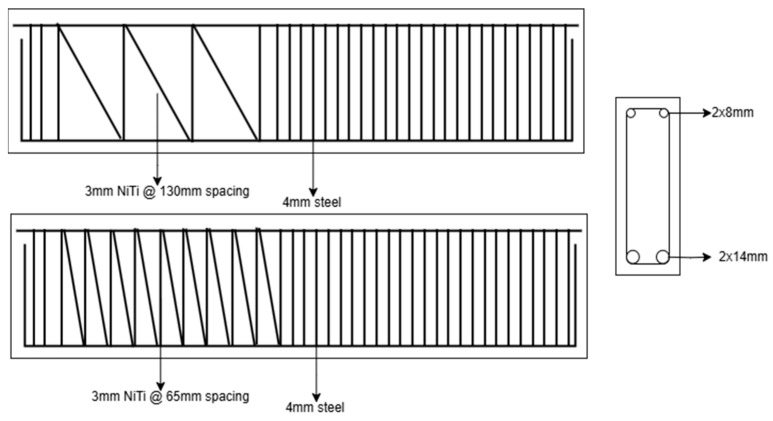
Beam geometries and different types of Ni-Ti reinforcement used [58].

NiTi wires are the third most used form of NiTi in civil engineering applications. They have been used as reinforcement in concrete beams. Li et al. [16] proposed a new method to strengthen RC structures through CFRP plates in combination with NiTi wires. The proposed method has been tested experimentally through an RC beam. The results demonstrated that the activation of NiTi wires reduced the deflection and could close cracks in concrete. In addition, a linear relationship was observed between the electric resistance of NiTi wires and mid-span deflection, which could lead to a potential method of damage detection in civil structures. Mas et al. [58] conducted an experimental trial to evaluate the possibility of obtaining a more ductile shear failure using NiTi wires. Seven concrete beam specimens have been reinforced with the spiral NiTi wires and tested to check the feasibility of this proposal, as shown in Figure 13. The results demonstrated that NiTi spiral reinforcement significantly increased the ductility and deformation capacity of concrete beams failing in shear.

In an innovative and remarkable experiment, Kuang and Ou [34] set up a smart self-repairing concrete beam using superelastic NiTi wires and a repairing adhesive. A static loading test was conducted on the concrete beam reinforced with NiTi wires and brittle fibers containing adhesives. The results indicated that NiTi wires enabled self-restoration and reversed the deflection of the beam, leading the cracks to close. Furthermore, the cracks were sealed after the adhesive-containing fibers broke and released the adhesive into the cracks in the first loading. During reloading, as the closed cracks remained sealed, the stress was redistributed to other uncracked sections where new cracks formed. Deng et al. [53] investigated how embedded NiTi wires in concrete beams could act as actuators. They examined various factors affecting the beam deflection, beam cross-sectional area, number of NiTi wires, pre-strain of NiTi wire, curing conditions and time, actuation mode, wire volume fraction, and diameter. They concluded that NiTi wires could produce notable recovery force and change the deflection of the beam when heated and used as actuators. Furthermore, Choi et al. [71] aimed to improve NiTi wires to exhibit self-centering and prestressing abilities in mortar beams. Various NiTi wire shapes were tested (as received, crimped, dog bone, and anchoring reinforced crimped) to maintain prestressing while achieving re-centering. Tests were conducted to assess crack closure and load bearing. Different heating methods were used to activate the phase transformation. Results displayed a drop in displacement recovery for as-received and dog bone wires, but similar ratios for crimped and anchoring reinforced crimped wires. Crimped wires heated electrically demonstrated comparable displacement recovery to superelastic SMA bars, indicating their potential for structural applications [72].

From the findings in the literature, it has been noted that NiTi wires can be used in columns’ confinement. El-Hacha and Abdelrahman [7] participated in two of these findings, as they conducted two experiments on column confinement using NiTi wires. One of the experiments studied how NiTi wires could influence the ductility and strength of RC columns actively confined with NiTi spiral reinforcement under eccentric loading. The results were compared with those of non-confined RC columns and those confined using CFRP sheets. The other paper focused on developing an analytical model to show how NiTi confinement enhanced the performance of an RC column under concentric uniaxial compressive loading. The model’s prediction was validated against experimental data, where it was concluded that the model could predict the column’s behaviour under such loading conditions [62]. Chen et al. [38] studied the active confinement of non-circular columns using NiTi wires. The specimens were tested under monotonic and cyclic uniaxial compression loads, and the results of NiTi-confined columns were compared with those of GFRP-confined columns. The conclusions drawn from the previously mentioned experiments [7,38] indicated that the active confinement of columns using NiTi wires could significantly improve concrete’s ultimate strain and residual strength. In addition, a more ductile response of columns has been noted along with control of crack opening and propagation and the ability to deform significantly before failure [7,38]. Moser et al. embedded NiTi wires in concrete to achieve prestress and prevent cracks in concrete. Specimens were heated to activate the NiTi wires and induce a prestress force. In conclusion, small prestress was obtained, and it was highlighted that further development in alloy selection and production methods was required to enable practical applications [6].

The literature on NiTi wires revealed certain limitations and areas requiring further research and development. A linear relationship was noted between the electric resistance of NiTi wires and the mid-span deflection. However, this correlation led to a potential method of damage detection. Accordingly, this application still requires further research before its widespread implementation by the industry. Furthermore, upon testing various NiTi wire shapes for self-centering and prestressing abilities in mortar beams, the results highlighted a decrease in wire displacement recovery, indicating that wire shapes were not equally effective for such purposes. Furthermore, another significant limitation is in the application of embedded NiTi wires for prestressing, in which small prestress was obtained. This led to the conclusion that further development in alloy selection and production methods was required to enable practical applications of embedded prestressing. Accordingly, this gap in the manufacturing process should be addressed, as it is hindering the widespread utility of NiTi wires for internal prestressing. It was also noted that the most effective strengthening of concrete elements was achieved by external prestressing with NiTi wires.

## 5. Mechanical and Thermomechanical Properties of NiTi

This section summarizes the NiTi’s mechanical and thermomechanical properties obtained from Table 2 and Table 3, which represent the mechanical properties of superelastic NiTi and the thermomechanical properties of SME NiTi, respectively. Superelastic NiTi has a high failure strain (28.6%), recovery strain (8%), yield stress (621 MPa), and ultimate strength (1483 MPa), whereas its elastic modulus (83 GPa) is in an intermediate range. Furthermore, another outstanding feature of superelastic NiTi is the ability to keep the residual stresses below 0.9% when recovering high amounts of strain. These notable properties aid in exhibiting perfect superelastic behavior, which promotes its replacement of conventional steel or uses along with traditional steel in specific areas where self-centering, strain recovery, residual stress, cyclic performance, and seismic performance are critical. The transformation phase stresses $\sigma_{s}^{AM}$, $\sigma_{f}^{AM}$, $\sigma_{s}^{MA}$, $\sigma_{f}^{MA}$ define the hysteresis loop of NiTi. The higher these values, the larger the hysteresis loop and the higher the strains a superelastic NiTi can recover. Similarly, SME NiTi has a high failure strain, recovery strain, yield stress, ultimate strength and an elastic modulus in the intermediate range.

Another important factor for the SME is recovery stress, which emphasizes the extent to which the SME of NiTi can recover from stress. A maximum value of 880 MPa for recovery stress is noted in Table 3, which is relatively high. The shape memory effect is better exhibited in SMAs, which have a wide thermal hysteresis between their martensite and austenite phases [85]. Subsequently, the phase transformation temperatures for SME NiTi are listed in Table 3. It can be noticed that the difference between martensite start temperatures and austenite start temperatures ranges from 30 to 35 °C, which is suitable for generating recovery stresses. However, a higher difference is more beneficial for generating higher recovery stresses [86,87]. Comparing both forms of NiTi, it can be seen that the elastic modulus of Super Elastic (SE) NiTi is higher, and the failure strain is lower. SE NiTi outperforms SME NiTi in terms of stiffness. This is attributed to the austenite phase, which has a high resistance to any externally applied stress due to its crystal structure, described as a body-centered cubic structure. In contrast, the crystal structure of the martensite phase is a parallelogram structure, leading to weaker resistance to externally applied stresses [5].

Past research has also shown that shape memory effect (SME) NiTi generally has intermediate activation temperatures, which causes a considerable drop in the recovery stresses generated due to the short-term relaxation when the heating process ends and the temperature drops. For an SMA to maintain its recovery stress, it should remain in the austenite phase, but this is not the case, as NiTi goes back to martensite as it cools down [85]. This barrier allows other alternatives like Ni-Ti-Nb and Fe-SMA to compete with it and perhaps outperform it in terms of generating prestressing energy or shape memory effect [85]. Nevertheless, both forms of NiTi remain significant and are a subject of interest nowadays for many researchers.

## 6. Conclusions

This paper presents a literature review on NiTi SMA, discussing its history, characteristics, forms, and shapes. Additionally, the incorporation of NiTi in concrete and cement-based composites and its effects have been considered by reviewing all NiTi-relevant papers in the literature, summarizing them into a table form, and briefly narrating their scope and findings. This review has demonstrated the significance of NiTi as a reinforcement or strengthening method and the potential of its utilization in concrete and cement-based composites more frequently in the form of bars, cables, wires, fibers, strands, or powder. The majority of the papers presented in this review have shed light on the possibility of replacing conventional steel rebars with NiTi bars, considering the need for further development to compensate for the lower modulus of elasticity of NiTi, which may result in reduced stiffness. However, many papers have highlighted promising hybrid reinforcement methods, including steel rebars and NiTi bars. Several outcomes and effects of NiTi on concrete have been reported in different papers, including enhanced ductility, less residual strain and stress, improved recovery and re-centering, better crack control and closure, enhanced energy dissipation, and improved corrosion resistance. With such advantages, it can be stated that NiTi can be a potential solution for structures exposed to earthquakes, blasts, natural phenomena, or structures of national importance. The most crucial findings and conclusions of the incorporation of NiTi in concrete are as follows:

- Improvement in flexural strength, compressive strength, modulus of elasticity, re-centering ability, toughness, peak load, energy absorption, delayed initial cracks, induced prestressing, improved bond resistance, and restricted crack width is owing to the prestressing effect of NiTi fibers upon incorporation in concrete.
- Less residual deflection leads to lower residual stress and higher recovery.
- Full recovery of strains and lower stiffness of NiTi bars in reinforced concrete; NiTi and high-strength steel provide reasonable stiffness and partial deformation recovery.
- Enhanced post-yield deformation recoverability, better seismic performance, and damage reduction.
- Stiffer beams with higher NiTi bar percentages show improved recovery ratios, reduced residual displacement, and higher cracking load with thinner NiTi bars.
- NiTi wires achieved prestress and exhibited self-centering abilities in beams.
- Ductile response with controlled crack opening, propagation, and significant deformation before failure.
- Increased NiTi fiber fraction and matrix compressive strength lead to higher flexural strength, toughness, deflection recovery, and crack closure.
- Crimped NiTi fibers are more efficient than other end-shape fibers.
- NiTi wires can generate a large recovery force when heated and can be used as actuators to adjust deflection in concrete beams.
- High ductility in shear and the ability to sustain significant load after critical shear crack development.
- NiTi bars provided effective prestressing and strengthening functions when used in concrete beams.
- Hybrid plastic hinge (steel + NiTi) frames increase lateral shear capacity, have better energy dissipation, and lower inter-story drifts while reducing construction costs compared to NiTi-reinforced frames.
- Crimped fibers with spearhead ends have the highest pullout resistance, while N-shaped end fibers have the lowest; increasing cross-sectional area or reducing the plateau can enhance functionality.
- Higher NiTi fiber content increases cyclic peak strength, reduces plastic strain, and improves peak strength and recovery stress; SME NiTi fibers provide bridging capacity.

## 7. Challenges and Future Prospects

Incorporating NiTi shape memory alloys (SMAs) in concrete potentially enhances the performance of concrete structures. Yet, its practical use is still limited by several challenges, including its high cost, limited availability, manufacturing challenges, and temperature sensitivity. Additionally, its long-term behavior, such as creep, durability, and fatigue, is not fully understood. In the absence of design codes for the use of SMA in concrete structures creates uncertainty in design approvals. Such challenges should be tackled prior to the wide adoption by the construction industry.

Additionally, while NiTi SMAs have been examined over the past twenty years, still much research is needed to fully understand its behavior in different civil engineering applications. For instance, future research should address the challenges posed by NiTi’s lower modulus of elasticity compared to steel. Hybrid reinforcement strategies, such as combining NiTi with other reinforcement materials, could mitigate this limitation or even yield an innovative member in terms of resilience and response to extreme conditions. Additionally, while using NiTi in reinforced concrete walls has shown promise, limitations in current designs and assumptions require further investigation. Studies investigating the behavior of NiTi-reinforced concrete walls under seismic events, long-term environmental impacts, and varying load conditions are necessary to refine design methodologies and expand their applications. Cost reduction and improved processing techniques for NiTi are essential to its adoption in large-scale projects. Long-term performance experiments under extreme environmental conditions, such as humidity, temperature variations, and chemical exposure, are vital for ensuring durability. Developing advanced simulation models, incorporating NiTi parameters into existing design software to predict the behavior of NiTi-reinforced systems and establishing standardized design guidelines will further accelerate its usage. Integrating academia and industry can position NiTi as a key element in future construction practices.

## Figures and Tables

**Table 2:** Minimum and maximum values of the mechanical properties of SE NiTi are reported in the collected data.

Property	Min Value	Max Value	Unit
*E*	18	83	GPa
$E_{py}$	1.7	2.2	GPa
$\sigma_{s}^{AM}$	300	621	MPa
$\sigma_{f}^{AM}$	335	700	MPa
$\sigma_{s}^{MA}$	73	370	MPa
$\sigma_{f}^{MA}$	32	187	MPa
$f_{u}$	195	1483	MPa
$\varepsilon_{u}$	0.1	0.286	%
$\varepsilon_{r}$	0.06	0.08	%
$\varepsilon_{res}$	0.0005	0.009	%

Note: $\sigma_{s}^{AM}$: Aust to Mart transformation phase start stress; $\sigma_{f}^{AM}$: Aust to Mart transformation phase finish stress; $\sigma_{s}^{MA}$: Mart to Aust transformation phase start stress; $\sigma_{f}^{MA}:$ Mart to Aust transformation phase finish stress; $\varepsilon_{r}$: Maximum recoverable strain; E: Modulus of elasticity; $f_{u}$: Ultimate stress; $\varepsilon_{u}$: Ultimate strain; $\varepsilon_{res}$: residual strain; $E_{py}$: post-yield stiffness; in superelastic NiTi, yielding stress = Aust to Mart transformation phase start stress.

**Table 3:** Minimum and maximum values of the thermomechanical properties of SME NiTi are reported in the collected data.

Property	Min Value	Max Value	Unit
*E*	8.7	52.5	GPa
$f_{u}$	818	1483	MPa
$f_{y}$	200	1248	MPa
$f_{r}$	200	880	MPa
$\varepsilon_{r}$	0.06	0.08	%
$\varepsilon_{u}$	0.106	0.65	%
$A_{s}$	30	85.54	°C
$A_{f}$	40	110.16	°C
$M_{s}$	−5	55	°C
$M_{f}$	−17	47	°C

Note: $\varepsilon_{r}$: Maximum recoverable strain; E: Modulus of elasticity; $f_{u}$: Ultimate stress; $\varepsilon_{u}$: Ultimate strain; $f_{y}$: Yield stress; $f_{r}$: maximum recovery stress; *A_s_*: Austenite start temperature; *A_f_*: Austenite finish temperature; *M_s_*: Martensite start; *M_f_*: Martensite finish temperature.

## Data Availability

No new data were created or analyzed in this study. Data sharing is not applicable to this article.
